# Immuno-modification of enhancing stem cells targeting for myocardial repair

**DOI:** 10.1111/jcmm.12439

**Published:** 2015-04-23

**Authors:** Jiashing Yu, Yuan-Kun Wu, Yiping Gu, Qizhi Fang, Richard Sievers, Chun-Hua Ding, Jeffrey E Olgin, Randall J Lee

**Affiliations:** a Department of Chemical Engineering, National Taiwan UniversityTaipei, Taiwan; b Department of Medicine, National Taiwan UniversityTaipei, Taiwan; c Division of Cardiology, Department of Medicine, University of California San FranciscoCA, USA; d Cardiovascular Research Institute, University of California San FranciscoSan Francisco, CA, USA

**Keywords:** stem cells, myocardial infarction, antibody targeting, electrophysiology

## Abstract

Despite the controversy in mechanism, rodent and clinical studies have demonstrated beneficial effects of stem/progenitor cell therapy after myocardial infarction (MI). In a rat ischaemic reperfusion MI model, we investigated the effects of immunomodification of CD 34^+^ cells on heart function and myocardial conduction. Bispecific antibody (BiAb), consisting of an anti-myosin light chain antibody and anti-CD45 antibody, injected intravenously was used to direct human CD34^+^ cells to injured myocardium. Results were compared to echocardiography guided intramyocardial (IM) injection of CD34^+^ cells and PBS injected intravenously. Treatment was administered 2 days post MI. Echocardiography was performed at 5 weeks and 3 months which demonstrated LV dilatation prevention and fractional shortening improvement in both the BiAb and IM injection approaches, with BiAb achieving better results. Histological analyses demonstrated a decrease in infarct size and increase in arteriogenesis in both BiAb and IM injection. Electrophysiological properties were studied 5 weeks after treatments by optical mapping. Conduction velocity (CV), action potential duration (APD) and rise time were significantly altered in the MI area. The BiAb treated group demonstrated a more normalized activation pattern of conduction and normalization of CV at shorter pacing cycle lengths. The ventricular tachycardia inducibility was lowest in the BiAb treatment group. Intravenous administration of BiAb offers an effective means of stem cell delivery for myocardial repair post-acute MI. Such non-invasive approach was shown to offer a distinct advantage to more invasive direct IM delivery.

## Introduction

Stem cell therapy has received considerable interest for the treatment of ischaemic cardiomyopathy 1,2. The notion of hematopoietic stem cell (HSC) transdifferention into new myocardium created initial excitement, but has been challenged with evidence supporting a paracrine effect and reduction in apoptosis, producing neovascularization and preserving LV function 3,4. Despite the controversy in mechanism, functional improvement after stem cell therapy has been reported 5–7. Moreover, the beneficial effect has been shown to be dose- and time-dependent, with a certain amount of cells required to observe beneficial results 8–10. These reports underscore some of the challenges in optimizing stem cell therapy, namely delivery, homing, engraftment, safety and efficacy.

We previously reported a bispecific antibody (BiAb) targeting strategy that created an organ-specific injury homing ‘molecule’ on the surface of CD34^+^ cells, resulting in improved CD34^+^ cell delivery and distribution to ischaemically injured myocardium, as well as induction of angiogenesis 1,11. The BiAb was engineered by chemical conjugation of a CD45 monoclonal antibody recognizing an antigen found in purified human CD34^+^ cells and a myosin light chain (MLC) monoclonal antibody recognizing a cardiac-specific antigen expressed in injured myocardium. As a cytosolic protein in cardiomyocytes, MLC will be exposed to intravascular anti-MLC only when the cell membrane is no longer intact, as would occur during myocardial injury. Thus, the CD45 x MLC BiAb armed cells can be anchored and uniformly distributed in the damaged myocardium. This less invasive approach has obvious advantages as a potential systemic delivery system for the delivery of bone marrow-derived stem cells to the myocardium, compared to direct intramyocardial (IM) or intracoronary injection.

To further explore the safety and efficacy of BiAb targeting of CD34^+^ cells, we investigated the effects of antibody targeted CD34^+^ cells on myocardial conduction following a myocardial infarction (MI) and its long-term effects on LV function.

## Materials and methods

### Myocardial infarction model

All surgical procedures were approved by the Committee for Animal Research of the University of California San Francisco (San Francisco, CA, USA). The ischaemia reperfusion model has been extensively tested in our laboratory 12–17. In brief, forty five female nude rats (200–250 g) were intubated endotracheally, connected to a rodent ventilator (Harvard Apparatus: Holliston, MA, USA), and anesthetized by inhalation of 2% isoflurane. A median sternotomy was performed, and the left anterior descending coronary artery was occluded with a single stitch of 7-0 Ticron suture for 25 min., and then reperfused. The chest was then closed, and the rat was allowed to recover.

### Conjugation for BiAbs, arming CD34^+^ cells and flow cytometry

The armed cells were prepared by chemically heteroconjugating the BiAbs on CD34^+^ cells. The conjugation of BiAbs has been reported previously 18. Briefly, mouse IgG1 anti-human CD45 (BD Pharmingen: San Jose, CA, USA) and mouse IgG2a anti-MLC (Abcam: Cambridge, MA, USA) were cross-linked with Traut’s reagent and SulfoSMCC (Pierce: Rockford, IL, USA), respectively, at room temperature for 1 hr. After eluting from desalting columns, the cross-linked antibodies were mixed at an equal molar ratio and heteroconjugated at 4°C overnight to produce anti-CD45 x anti-MLC (CD45 x MLC).

CD34^+^ cells (Cambrex, now Lonza: Allendale, NJ, USA) were armed with CD45 x MLC by incubating 15 min. at room temperature, washed twice, spun down and resuspended in PBS. The arming concentration for the *in vivo* study was 0.5 μg/million cells. CD45 x MLC-armed CD34^+^ cells were incubated with antimouse IgG2a+b PE (BD 340269, 20 μl/test, 0.0015 mg/ml) or IgG isotype control antibodies and then subjected to flow cytometry to detect PE positive cells (anti-CD45 positive; BD FACSCalibur: Becton Dickinson, San Jose, CA, USA).

### Administration of CD34^+^ cells

Based on fractional shortening (FS) 2 days after myocardial injury, 30 rats were categorized into 6 barely damaged (35% < FS < 45%), 18 damaged (25% < FS < 35%) and 6 severely damaged (FS <25%). Then, the 30 rats were randomized into three groups, each consisting of two barely damaged, six damaged and two severely damaged animals. The groups consisted of: (*i*) direct injection of unarmed CD34^+^ cells (2 million cells in 0.50 cc of PBS), (*ii*) intravenous injection of BiAb CD45 x MLC armed CD34^+^ cells (2 million cells in 0.25 cc of PBS) and (*iii*) intravenous injection of PBS as a control (0.25 cc). Each group consisted of 10 animals. One rat was intravenously injected with BiAb CD45 x MLC armed iron oxide-labelled CD34^+^ cells and killed 1 day afterwards to verify the successful homing.

Intravenous injections were administered *via* the jugular vein when rats were anaesthetized by isofluorane. Direct IM injections were guided by high-resolution echocardiography 19. Briefly, echocardiography was performed with the Vevo 660 system (VisualSonics, Toronto, ON, Canada). A 50 μl Hamilton syringe loaded with unarmed CD34^+^ cells in 50 μl PBS was secured in a micromanipulator and aligned with the ultrasound probe such that the needle could be viewed under echocardiography. The needle was advanced with the micromanipulator through the body wall and diaphragm until the needle level was in the centre of the hypokinetic part of the LV free wall under the echo guidance. The unarmed cells or PBS only was injected in this injured region.

### Echocardiography

Transthoracic echocardiography was performed with a 15-MHz linear array transducer system (Sequoia c256; Acuson: Malvern, PA, USA) on all animals under anaesthesia by inhalation of 1.5% isofluorane as previously described 10,12. Echocardiography was performed 1 day after MI (prior to therapy), then 10–12 days, 5 weeks and 3 months after therapy. The echocardiographer was blind to the treatment groups during data acquisition and analysis. Two-dimensional images were obtained in both parasternal long- and short-axis views at the papillary muscle level. M-mode images were obtained, and the wall thickness and LV internal dimensions (LVD) were measured. Fractional shortening as a measure of systolic function was calculated from LVD in diastole and systole (FS = (LVDd − LVDs)/LVDd × 100%).

### Histology

Sixteen weeks after infarction, the rats were killed with a pentobarbital overdose (200 mg/kg) and the hearts were immediately excised and fresh-frozen in O.C.T. freezing medium (Sakura Finetek: Torrance, CA, USA). The hearts were then sectioned into 10-μm slices throughout the infarct region, and the representative slices were stained with Masson’s trichrome stain for morphometric analysis.

Six sections equally distributed throughout the infarct area were stained with mouse IgG1 anti-human leucocyte antigen class I (BD Pharmingen: San Jose, CA, USA) to detect the CD34^+^ cells.

Angiogenesis in the infarct was examined by immunohistochemical staining of CD31 (mouse IgG1 anti-CD31; BD Pharmingen 550300) and α-smooth muscle actin (mouse IgG2a anti-α smooth muscle actin; Sigma-Aldrich: St. Louis, MO, USA) to detect capillaries and arterioles respectively. The staining assay was then finished with a mouse-on-rat polymer kit (Biocare: Concord, CA, USA). Capillaries were identified as a single layer of CD31-positive cells, and capillary density was obtained from five high-power fields in the infarct region on each slide and averaged over the slides spanning the entire infarct. Arterioles were identified as a positive staining of α-smooth muscle actin with a lumen structure of diameters between 10 and 100 μm. Arteriole density was calculated as the average number of arterioles in the total infarct area from six representative slides 10. The infarct size was calculated as the infarct area percentage of the left ventricle area 10.

### Assessment of fibrosis pattern

To differentiate the MI pattern in the three groups, we developed a method to semi-quantify the fibrosis pattern in different regions 13–15. Up to five evenly distributed views within the MI were selected for each slide and up to 12 slides per animal with Masson’s trichrome stain were scored on a 1 to 4 scale. The scale was defined as: (*i*) infarct area consisting of greater than 90% dense scar, (*ii*) infarct area consisting of less than 90% but greater than 50% dense scar, (*iii*) infarct area consisting of less than 50% but greater than 10% dense scar and (*iv*) infarct area consisting of less than 10% dense scar. Given the fact that up to 60 scores were averaged as a fibrosis pattern score for each animal, we assumed a normal distribution for the averaged fibrosis pattern score although the scores are more categorical than continuous.

### Optical mapping

Optical Mapping was performed 5 weeks after cell treatment as previously described 20. Two rats from each group received 500 U heparin intraperitoneally 15 min. before being anesthetized with pentobarbital (50 mg/kg). The heart was rapidly harvested, cannulated through the aorta and perfused at 6 ml/min. with 37°C modified-Tyrode solution consisting (in mM) of 130 NaCl, 20 NaHCO_3_, 1.2 NaH_2_PO_4_, 1.0 MgCl_2_, 4.0 KCl, 5.6 dextrose and 1.8 CaCl_2_ (pH 7.4). The modified-Tyrode solution was bubbled with 95% O_2_ and 5% CO_2_. The heart was then transferred to a custom-built optical mapping chamber and 15 mM of the excitation-contraction uncoupler 2,3-butanedione monoxime and 15 mM of the voltage-sensitive dye Di-4-ANEPS were added to the modified-Tyrode solution.

Light from a 1000 W tungsten-halogen lamp (Model 66921; Spectra-Physics: Santa Clara, CA, USA) *via* fiberoptic cables was directed onto the field of view for illumination. Care was taken to illuminate the imaging surface homogeneously. Optical mapping images of 100 × 100 pixels were obtained with an excitation filter of 530 nm and emission long-pass filter with a cut-off of 630 nm. Images were acquired by an ultra-fast imaging system MICAM ULTIMA (Scimedia: Costa Mesa, CA, USA) at 1000 Hz.

### Pacing protocol, effective refractory period and data analysis

The perfused hearts underwent ventricular epicardial bipolar pacing for 20 cycles at pacing cycle lengths (PCL) of 200, 180, 150 and 130 msec., and then decreased by 10 msec. until loss of capture. Programmed electrical stimulation consisting of a S1-S2 protocol was applied with a basic PCL of 200 msec. for 20 cycles and S2 from 150, 130 and 120 msec., and then decreased by 10 msec. until the effective refractory period (ERP). Burst pacing was applied from 100 to 60 msec. by decreasing intervals of 2 msec. in an attempt to induce ventricular arrhythmias. Non-sustained ventricular tachycardia (VT) was defined as 3 or more consecutive ventricular ectopic beats lasting less than 30 sec. Sustained ventricular tachyarrhythmias were defined as sustained VT ≥30 sec. or ventricular fibrillation.

Quantitative analysis was performed by identifying activation of each optical action potential as the maximum dF/dt. Isochronal activation maps, conduction velocity (CV), velocity vectors 21, and rise time (time between takeoff and peak of the action potential) were analysed 20,22. Action potential duration was determined as APD50 and APD80 (duration from activation to the point at which the amplitude had recovered to 50% and 80% of its maximum respectively).

### Statistics

anova was carried out for comparisons between groups. Unpaired two-tail *t*-tests were applied for comparison between MI and normal tissue in each animal in echocardiography, vessel density and fibrosis pattern results. Statistical significance was considered to be a *P*-value of less than 0.05.

anova was performed for comparisons between groups to analyse the echocardiography data. The *P*-value was reported using an unpaired two-tail *t*-test.

Animals for optical mapping were randomized and paired two-tail *t*-tests were applied for comparison between MI and normal tissue in each animal while anova followed by an unpaired two-tail *t*-test was applied for comparison between groups.

## Results

### CD45 x MLC BiAb binding kinetics

**Figure 1:**
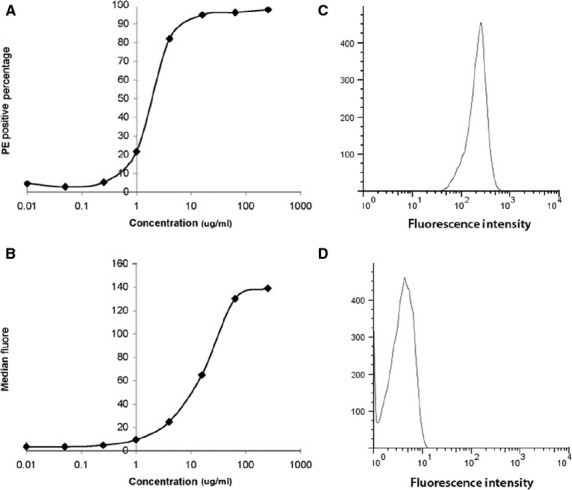
The optimal arming BiAb concentration is decided based on BiAb binding efficiency. The percentage of cells with a positive stain of CD45xMLC BiAb increases as the BiAb concentration increases during arming, and 95% cells are positive at concentrations higher than 16 μg/ml (A), while the median fluorescence intensity is saturated at higher concentration (greater than 64 μg/ml (B), because a positive stain of MsIgG2a indicates the binding of BiAb but not saturation of cell surface binding sites (CD45). The cells armed at 8 μg/ml are almost all positive in MsIgG2a (C) compared to the unarmed cells (D).

The BiAb binding efficiency on CD34^+^ cells is dose-dependent. Percentage of mouse IgG2a (isotype of anti-MLC) positive cells increases as BiAb concentration increases and plateau at concentrations greater than 16 μg/ml at which 95% cells are positive with anti-MLC (Fig.1A). The median fluorescence intensity plateaus at about 100 μg/ml (Fig.1B). This higher plateau concentration is because of the surface binding site of a cell, CD45, is not saturated with BiAb CD45 x MLC when the cell carried enough mouse IgG2a exceed the threshold of positive PE staining of mouse IgG2a so that a PE positive cell will have more fluorescence emission when the BiAb concentration increases.

This result gave a guideline of arming concentration for *in vivo* studies. CD34^+^ cells were armed at 0.5 μg/million cells at 16 million cells/ml, as this concentration gave higher binding efficiency and minimized the potential side effect of BiAb. Flow cytometry after cell injection showed that more than 90% CD34^+^ cells are armed with CD45 x MLC BiAb (Fig.1C) while the unarmed cells are all negative in mouse IgG2a (Fig.1D).

### BiAb homed CD34^+^ cells preserve heart function comparable to direct injection

**Figure 2:**
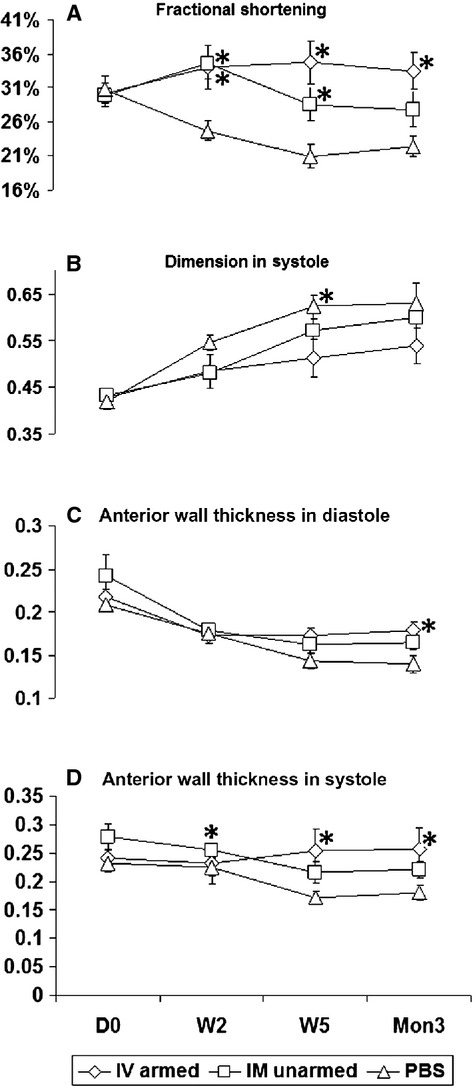
Echocardiography data show functional improvement in treatment groups, and the group treated with armed cells intravenously (IV armed) shows similar improvement to the group treated with direct injection cells into the myocardium (IM direct) at different time-points after cell injection (D0 is the baseline before treatment). All the data are represented as mean + SEM, with stars representing statistical significance compared to the PBS group (*P* < 0.05). *indicates statistically significant compared with PBS control groups.

The baseline measurements (before cell injection, D0), including FS, LVD, LV anterior wall thickness (AWT) in systole and diastole, showed no difference among the three groups (Fig.2). The PBS control group showed the typical negative remodelling following a MI, which showed left ventricle function deterioration, LV dilation and LV free wall thinning. In contrast, the groups treated with CD34^+^ cells demonstrated improved LV function and attenuated wall thinning. As early as 2 weeks, FS in both BiAb armed (34.02 ± 10.33%, *P* = 0.017) and direct injection groups (34.54 ± 7.41%, *P* = 0.002) increased significantly compared to the control group (24.70 ± 4.36%). This significant improvement persisted for 5 weeks in both the BiAb armed group (34.69 ± 10.04%, *P* = 0.001) and the direct injection group (28.54 ± 7.78%, *P* = 0.021), compared to the control group (20.97 ± 5.46%). However, after 3 months, only the BiAb armed group (33.48 ± 8.48%, *P* = 0.002) was significantly better than the control group (22.39 ± 4.63%). The systolic LV dimension in the BiAb armed group (0.512 ± 0.125 cm) was significantly (*P* = 0.013) less than that in the PBS control group (0.623 ± 0.08 cm), whereas the systolic LV dimension in the direct injection group trended towards significance (0.571 ± 0.08 cm, *P* = 0.052) at week 5. In both systole and diastole, LV wall thinning was retarded in the treatment groups and more significant in the BiAb armed group (*P* < 0.05) than in the direct injection group (*P* < 0.1), compared to the PBS control group (Fig.2C and D). Anterior wall thickness in systole (AWTs) in the PBS control group were 0.226 ± 0.049 cm, 0.172 ± 0.038 cm, and 0.180 ± 0.044 cm at 2 weeks, 5 weeks and 3 months respectively. AWTs in BiAb armed group were 0.232 ± 0.059 cm (*P* = 0.018), 0.253 ± 0.056 cm (*P* = 0.001) and 0.257 ± 0.056 cm (*P* = 0.004) and AWTs direct injection group were 0.255 ± 0.042 cm (*P* = 0.015), 0.216 ± 0.059 cm (*P* = 0.068), and 0.222 ± 0.046 (*P* = 0.078) at 2 weeks, 5 weeks and 3 months respectively. AWTs in the BiAb armed group were significantly thicker than that in control group at the corresponding time-points while AWTs in the direct IM injection group was only significant at 2 week. Anterior wall thickness in diastole (AWTd) in the BiAb armed group (0.178 ± 0.032 cm) was significantly (*P* = 0.01) thicker than that (0.139 ± 0.032 cm) in PBS control group only at 3 month and AWTd in was not significant in the direct injection group at 3 month (0.165 ± 0.027 cm; *P* = 0.056).

### Human CD34^+^ cells treatments reduce infarct size and MI has different patterns

**Figure 3:**
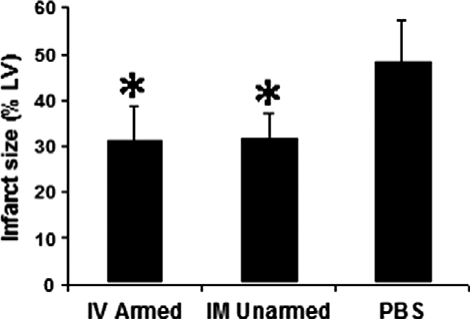
Infarct size. The IV injection of armed CD34^+^ cells resulted in similar infarct size to that of the conventional (IM unarmed) approach. Both reduced infarct size significantly (*P* < 0.05) compared to injection of PBS. Data are represented as mean + SD. *indicates statistically significant compared with PBS control groups.

After 3 months, no human cells could be detected with immunohistochemistry staining. Infarct size (the percentage of infarct area to LV area) showed similar results (Fig.3) as the echocardiography data. The BiAb armed group had similar infarct size (31.12 ± 7.7%) to the direct injection group (31.51 ± 5.9%); and both were significantly smaller than the PBS control group (48.04 ± 9.2%; *P* < 0.001).

**Figure 4:**
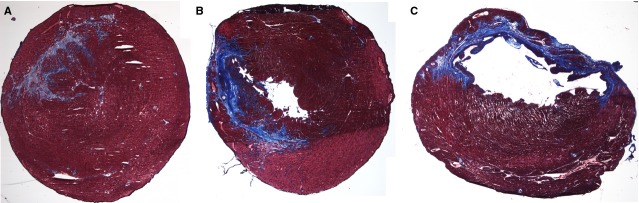
Masson’s trichrome staining of three slides with infarct sizes close to the average infarct size in the IV armed group (A) (28.49%), direct injection group (B) (33.19%), and PBS control (C) (43.44%). The infarct pattern is denser in the CD34^+^ direction injection group than in the IV armed group, and more transmural infarcts are observed in the PBS control group.

**Figure 5:**
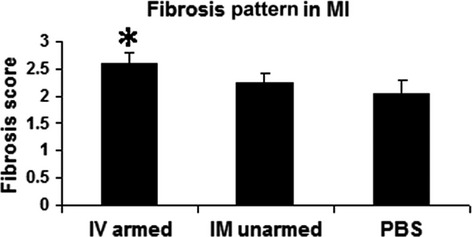
Fibrosis Pattern in MI. Fibrosis score were averaged within each group. The group with IV injection of armed CD34^+^ cells has a significantly higher score: in other words, a more patchy pattern in the MI, than in the PBS group but not in the IM unarmed group. *indicates statistically significant compared with PBS control groups.

Although the two treatment groups had similar infarct size, the scar in the BiAb armed group (Fig.4A) was more patchy than that in the IM unarmed group (Fig.4B). Transmural infarcts were present more often in the PBS control group (Fig.4C). We further differentiated the difference of fibrosis pattern among groups as described in the method section. The averaged score of the BiAb armed group (group averaged score = 2.59) was significantly higher (*P* < 0.01) than the PBS control (group averaged score = 2.06) but not significant in the IM unarmed group (group averaged score = 2.24, *P* = 0.11). This suggests that scar in the BiAb armed group were more patchy (Fig.5).

### BiAb targeted CD34^+^ cells promote angiogenesis and uniform neovascularization

**Figure 6:**
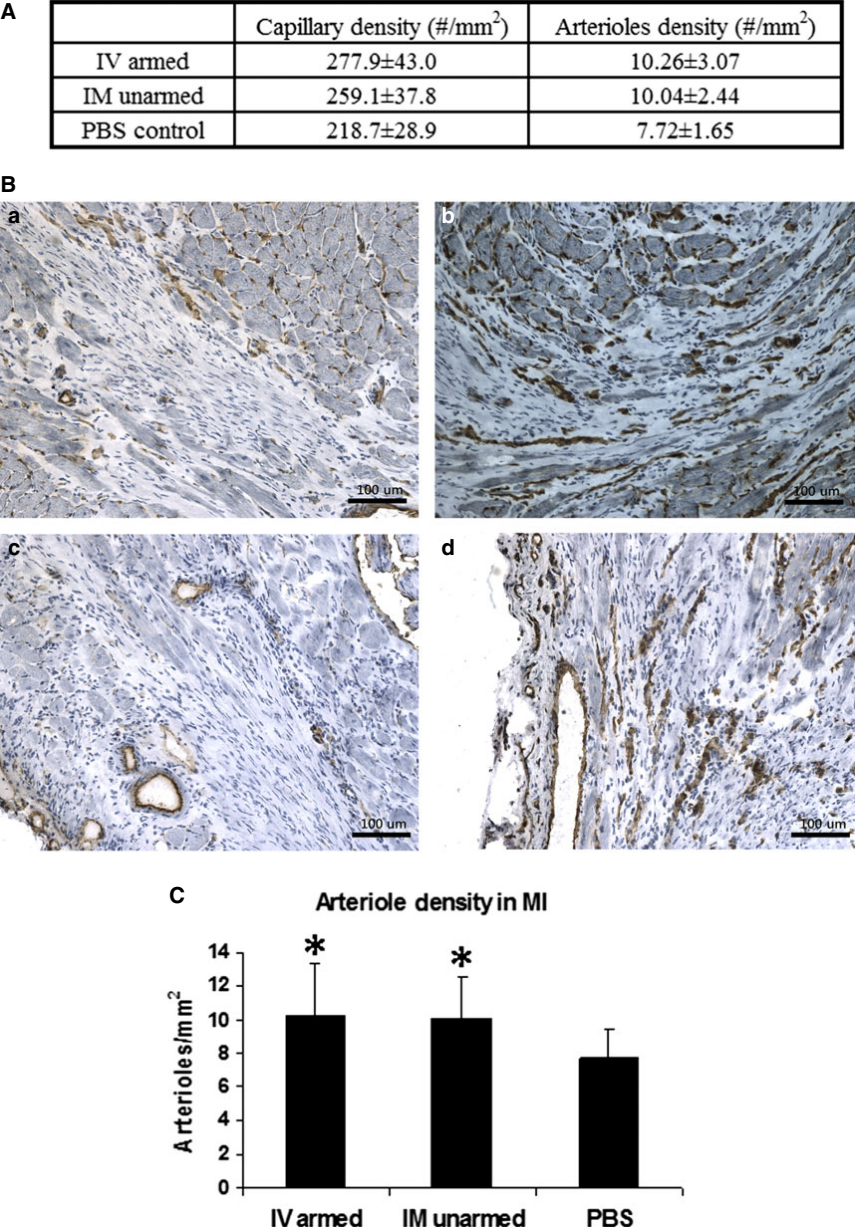
(A) Angiogenesis data (B) Staining of capillaries at the border of the MI in PBS (a), BiAb (b) and direct injection groups (c, d). Capillary density in direction group varies greatly at different sites and can be as sparse as the PBS group (c) or as dense as the BiAb group (d). (C) Arteriole density. The IV injection of armed CD34^+^ cells and direct injection of CD34^+^ cells increase the arteriole density significantly (*P* < 0.05) compared to injection of PBS. Data are represented as mean + SD. *indicates statistically significant compared with PBS control groups.

Angiogenesis was assessed by determining the capillary and arteriole density. CD31 staining for capillaries and α-smooth muscle actin staining for arterioles revealed an increase in average capillary and arteriole density in the CD34^+^ treated groups (Fig.6, mean ± SD). Capillary densities in the BiAb armed group (*P* = 0.01) and the direct injection group (*P* = 0.04) were significantly higher than that of the PBS control group. No significant difference in arteriole density was noted. Representative pictures of CD31 staining were selected (Fig.6) to demonstrate that there are fewer capillaries in the infarct region of the PBS control group (Fig.6A) than in the BiAb (Fig.6B) targeted group. The capillary density in the direct injection group had a larger variance which depended on the location within the MI and can be just as sparse as the PBS group (Fig.6C) and as dense as or denser than the BiAb targeted group (Fig.6D) in the same animal. This might be explained as the proximity to the injection site (the infarct area). The average arteriole densities in BiAb armed group and direct injection group were higher than the PBS group (Fig.6).

### BiAb targeted CD34^+^ cells restores electrophysiological properties of the MI region

**Figure 7:**
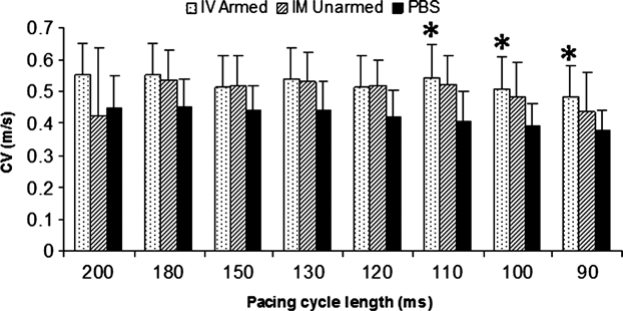
Conduction velocities in MI in the three groups. Conduction velocity in MI was significantly larger in the IV armed group than in the PBS control group at PCLs of 110, 100 and 90 msec. Data are represented as mean + SD. *P < 0.05 compared to the PBS control.

Conduction velocity, APD, and rise time of the normal ventricular tissue were similar among the groups (data not shown). However, differences in the MI regions among the groups were noted. Average CV in the BiAb targeted group, but not the direct injection group, was significantly faster than that in the PBS control group at PCL 110, 100 and 90 msec. in MI (Fig.7) and approached normal value. However, both BiAb armed and IM unarmed groups had shorter mean APD80 and APD50 than the PBS control group in the MI region. Only APD50 at PCL 200 msec. in the BiAb armed group was significantly shorter than that in the PBS control group.

**Figure 8:**
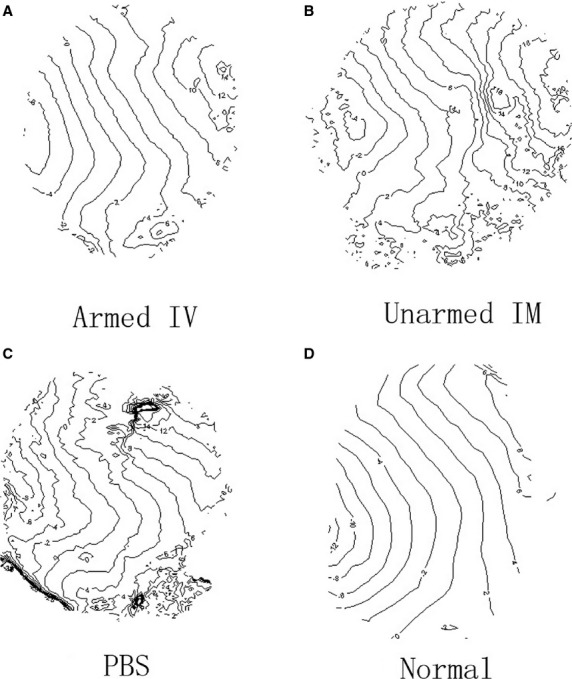
Isochromal maps at a pacing cycle length of 200 msec. Isochronal maps were reconstructed from representative animals with different treatments: BiAb armed CD34^+^ cells (A), direct injection of unarmed cells (B), injection of PBS (C) and normal heart without MI (D). Lines in all the maps are 2 msec. apart.

Conduction patterns were also different among the groups. Isochronal maps at PCL 200 msec. were plotted (Fig.8) from one representative animal in each group (Fig.8A–C), and an isochronal map from a healthy rat without an MI paced also from the left side of the ventricle at 200 msec. was listed as a reference (Fig.8D). The isochronal map in the BiAb armed group (Fig.8A) demonstrated relatively slower conduction through the MI located around the upper left centre of the map. The conduction map of the BiAb armed group was more uniform compared to the IM unarmed and PBS control groups and more similar to that of a normal heart (Fig.8D). In contrast, the isochronal map in the IM unnamed group (Fig.8B) showed a dramatic decrease in conduction through the MI located at the right centre, while the conduction in the PBS control group (Fig.8C) demonstrates a block within the MI region.

### Effective refractory period

There was no significant difference in ERP between the three groups. The ERP was 56 msec. in the BiAb armed group, 58 msec. in the IM unarmed group, and 64 msec. in the PBS control group.

### Ventricular tachycardia inducibility

In the PBS control group, one out of five rats had sustained VT and two rats had non-sustained VT (60%). Two out of five rats had non-sustained VT in the IM unarmed group (40%), and one out of five had non-sustained VT in the BiAb armed group (20%).

## Discussion

This study demonstrated the long-term effectiveness of antibody targeting technology in stem cell therapy for myocardial repair following a MI. The beneficial effects were sustained improvement in LV function, increased angiogenesis and a trend towards normalization of myocardial conduction. The noninvasive intravenous delivery of CD34^+^ cells resulting in a more uniform repair of infarcted myocardium offers a distinct advantage over the more invasive direct IM delivery. The BiAb technology provides a ‘homing molecule’ to target potential therapeutic agents to specific myocardial injury antigens.

Despite the improvements in LV function, increased angiogenesis and decreased infarct size were similar in both the BiAb armed and echo guided direct injection groups. Histological findings suggested more uniform repair within the infarct area of the BiAb armed group. The neovascularization in the BiAb armed group was seen throughout the infarct area. In contrast, the echo guided direct injection of the CD34^+^ group demonstrated a wide variance of neovascularization within the infarct area (Fig.6). There were some areas of the infarct with little to no neovascularization resembling the placebo control, while other infarct areas clearly had neovascularization within the infarct area. Furthermore, a qualitative assessment of the MI scar suggests that the BiAb armed group has a less dense scar compared to both the echo guided direct injection of CD34^+^ cells and control groups.

The more uniform infarct pattern of the BiAb armed CD34^+^ group may be the result of the CD34^+^ cells being brought to the MI *via* circulation, leading to a higher number of CD34^+^ exposed to the border and centre of the MI, where part of the vasculature is still functional and a more favourable milieu exits for HSCs, thus allowing the BiAb to bind to the MLC and deliver the CD34^+^ cells to areas of injury. In contrast, direct injection is a less controlled delivery that may result in high concentrations of cells in parts of the MI but an inhomogeneous distribution within the MI. The difference in the distribution of the two treatments may explain the similar improvements but possible different patterns of angiogenesis and scar density in the BiAb armed and echo guided treatment groups. This is also consistent with our finding of a more uniform CV pattern within the MI of the BiAb armed group compared to the echo guided direct injection and control groups. Further biodistribution and pharmacokinetic studies are required to determine the homing efficiency and to allow for a more complete comparison to direct injection methods. Moreover, with the recent discovery of telocytes in endocardium and epicardium, interaction of BiAb, CD34^+^ cells and telocytes require further investigation 23,24.

Unlike our previous study which demonstrated persistence of CD34^+^ cells 5 weeks after HSC injection 18, no HSCs were seen 3 months after injection. This agrees with other clinical studies that suggest the beneficial effect may be explained by the paracrine effect of HSCs. The secretion of HSCs may have modified the milieu in the MI, induced angiogenesis, helped the survival of cardiomyocytes, and further retarded the negative remodelling process. The transplanted HSCs may undergo apoptosis or be removed in the long run, but the beneficial effect is sustained for at least 3 months.

The optical mapping studies demonstrated that (*i*) electrophysiological properties were significantly changed within the MI, (*ii*) CD34^+^ cell treatment of MI was not arrhythmogenic and (*iii*) the CD45 x MLC armed CD34^+^ cell group showed significantly better electrophysiological properties within the infarction than the control treated group. Within the MI, CV was more than 20% slower, APD was 10% longer, especially with longer PCL and the rise time was 20% longer compared to normal tissue. Ventricular tachycardia inducibility did not increase in the animals that received CD34^+^ cells, and in fact decreased in the IV armed group (*P* = 0.08704), although not significantly. CD34^+^ cell treatment did not affect CV in the normal tissue but in the MI, CV was significantly faster in the BiAb armed group than in the control group at shorter PCL. Therefore, the averaged CV was relatively closer to CV in normal tissue. Action potential duration in MI was significantly shorter in the BiAb armed group than in the control group at a PCL of 200 msec. These findings proved that treating MI with BiAb armed CD34^+^ cells was not pro-arrhythmic, consistent with other reports 25 and tended to normalize conduction. These beneficial effects on electrophysiology in the BiAb armed group were likely secondary effects from the prevention of negative remodelling, improvement in blood supply in the border zone, and preserved cardiac function, rather than a direct effect of CD34^+^ cells coupling with cardiomyocytes and enhancing coupling. Previous observations have shown a low transdifferentiation rate of the transplanted CD34^+^ cells 1,18.

Combined with previous reports demonstrating that BiAb targeted HSCs improve LV function 1,18, we conclude that antibody targeting approach provides a non-invasive, efficient, and effective delivery method for cardiac stem cell therapy. The more uniform distribution of CD34^+^ cells in the BiAb armed group leads to a more uniform repair pattern of angiogenesis and myocardial conduction.
